# Privacy-Preserving Authentication Using a Double Pseudonym for Internet of Vehicles

**DOI:** 10.3390/s18051453

**Published:** 2018-05-07

**Authors:** Jie Cui, Wenyu Xu, Hong Zhong, Jing Zhang, Yan Xu, Lu Liu

**Affiliations:** 1 School of Computer Science and Technology, Anhui University, Hefei 230039, China; cuijie@mail.ustc.edu.cn (J.C.); xwysml@163.com (W.X.); root_zj@163.com (J.Z.); xuyan@ahu.edu.cn (Y.X.); 2 Anhui Engineering Laboratory of IoT Security Technologies, Anhui University, Hefei 230039, China; 3 Department of Computing and Mathematics, University of Derby, Derby DE22 1GB, UK; l.liu@derby.ac.uk

**Keywords:** batch verification, IoV, TPD, privacy-preserving authentication

## Abstract

The Internet of Vehicles (IoV) plays an important role in smart transportation to reduce the drivers’s risk of having an accident and help them manage small emergencies. Therefore, security and privacy issues of the message in the tamper proof device (TPD) broadcasted to other vehicles and roadside units (RSUs) have become an important research subject in the field of smart transportation. Many authentication schemes are proposed to tackle the challenges above and most of them are heavy in computation and communication. In this paper, we propose a novel authentication scheme that utilizes the double pseudonym method to hide the real identity of vehicles and adopts the dynamic update technology to periodically update the information (such as member secret, authentication key, internal pseudo-identity) stored in the tamper-proof device to prevent the side-channel attack. Because of not using bilinear pairing, our scheme yields a better performance in terms of computation overhead and communication overhead, and is more suitable to be applied in the Internet of Vehicles.

## 1. Introduction

In recent years, the Internet of Vehicles (IoV) [1,2,3,4] has aimed to enhance driving safety through inter-vehicle communications and communications between vehicles and roadside infrastructure [5,6,7]. Both academia and industry show great interests in developing a secure and efficient IoV. A typical IoV consists of a trusted third party (TA), a set of Roadside Units (RSUs) distributed along the roads, and many vehicles driving on the road. In IoV, the RSUs communicate with the TA via wired connections, and communicate with the vehicles via wireless channels. A vehicle periodically broadcasts traffic safety related messages such as the speed of the vehicle, the road condition, etc., to nearby vehicles and RSUs using the Dedicated Short Range Communications (DSRC) [8] protocol. These messages can be helpful to deal with emergency road conditions and reduce the risk of accidents.

After receiving messages sent by a vehicle, the RSU or vehicle needs to verify the integrity of the message to ensure that it is not modified by the attacker during the transmission. Meanwhile, the real identity of the vehicle should not be known by a malicious attacker during the transmission to preserve the identity privacy of the sender. However, false messages from attackers may cause significant damages; therefore, for security concerns, a trusted third party is needed to retrieve the real identify and locate the attackers who send the false messages. Many efforts have been made to tackle the above challenge, and many authentication schemes including Chim [9] have been proposed. Most of them are heavy in computation and communication.

To reduce the computation and communication overhead of the existing authentication scheme, in this paper, we propose the novel privacy-preserving authentication using a double pseudonym for the Internet of Vehicles. Our scheme makes use of the double pseudonym method and dynamic update technology. The computation and communication overhead are reduced because no bilinear paring is needed in the signature generation and verification. In addition, we show that the proposed scheme is secure via comprehensive security analysis. Finally, we periodically update the informations (e.g., member secret, authentication key, IPID) stored in the tamper-proof device; therefore, our scheme can resist the side-channel attack.

The remainder of this paper is organized as follows. Section 2 shows the related work about the identity-based scheme for IoV. The system model and security requirements are presented in Section 3. We describe the design of our scheme in Section 4 and the security analysis of the proposed scheme is indicated in Section 5. Section 6 analyzes the computational overhead and the communication overhead of the proposed scheme. Finally, conclusions and future work are presented in Section 7.

## 2. Related Work

Security and Privacy issues have attracted wide attention in IoV. Based on our knowledge, there are three types of authentication methods, namely, an anonymous certificate authentication scheme based on Public Key Infrastructure (PKI) [10], a group authentication scheme based on group signature, and a signature verification scheme based on identity. In 2006, Gamage et al. [11] used an identity-based ring signature scheme to protect the true identity of the signer. However, it was not possible to retrieve the true identity of the sender when the message was disputed. Later, Raya and Hubaux [12] proposed a PKI-based authentication scheme to achieve privacy preserving. Firstly, in order to protect the real identity of the vehicle, every vehicle needs to pre-load a large number of public and private key pairs and the corresponding certificate, which caused a serious storage management burden for a vehicle. Secondly, the trusted third party (TA) also suffers from a heavy certificate management burden to maintain all the anonymous certificates of all the vehicles. Furthermore, when the RSU or vehicle checks the validity of the signature, it is necessary to check the validity of the corresponding certificate, which also causes additional overhead to the system.

Some group-based signature schemes [13,14,15] were also proposed in the same year, where the group manager holds the private key of the group and can restore the true identity of the message signed by any vehicle in the group. In Lin’s scheme [14], many vehicles form a group in which each vehicle has its own private key and shares a group of public keys. They use a group signature to implement anonymous authentication of messages sent from a vehicle, and to use identity-based signatures to ensure the integrity of the messages sent from the RSU. A vehicle generates a signature of the corresponding message with its own private key, and the adversary could not link two anonymous identities or two signatures generated by the same vehicle. Hence, the proposed scheme provides unlinkability. Although a traditional certificate management problem is avoided in the group signature-based authentication scheme, the size of the CRL (Certificate Revocation List) grows as the number of recovered signers increases. Each CRL checking operation involves two pairing operations, which results in serious computational overhead for signature verification.

In order to neutralize the above two schemes, in 2008, Zhang et al. [16] first proposed an identity-based batch authentication scheme using a bilinear mapping. Firstly, in Zhang’s scheme [16], a large number of public and private key pairs and the corresponding certificate do not need to pre-load into a vehicle, which greatly reduces the overhead of transmitting and verifying the public key certificate. Secondly, the scheme uses the batch authentication method to verify the many messages at the same time, which can reduce the computation overhead. Thirdly, since a vehicle uses a pseudonym identity attached to the message during the transmission process, some untrustworthy parties and malicious attackers could not know the real identity of the vehicle. Finally, when a false message is found, a trusted third party has the ability to reveal the true identity of a vehicle. Therefore, the conditional privacy protection could be achieved.

However, in 2013, Lee and Lai [17] pointed out that Zhang’s scheme [16] had some flaws. First of all, Zhang’s scheme [16] cannot resist replay attack. In the absence of the corresponding inspection device, the receiver maybe receive a valid signature that has been verified before. Secondly, Zhang’s scheme [16] could not achieve non-repudiation. Although a trusted third party (TA) could recover the real identity of a false message that is sent by an adversary, the attacker could also deny sending the corresponding message. Hence, Lee and Lai [17] proposed an improved scheme to achieve better privacy preserving.

Recently, Zhang et al. [18] and Bayat et al. [19] found that Lee and Lai’s scheme [17] was not able to resist impersonation attacks, that is, malicious attackers could simulate a legal vehicle to send false messages. Therefore, Zhang et al. [18] and Bayat et al. [19] proposed two improved schemes to address the problem in Lee and Lai’s scheme [17]. However, as pointed out in He et al.’s scheme [20], the two schemes above have flaws in that they cannot prevent the modification attack in which the signature of message could be modified by the malicious attacker. Therefore, He et al. [20] proposed a conditional privacy protection scheme that does not use bilinear paring.

In He et al.’s scheme [20], since the system’s master private key is stored in a tamper-proof device (TPD), which is a device from which that no attacker can extract any stored data, the attacker could not acquire the system’s master private key to control the whole system. However, in a side-channel attack, the adversary collects a side channel information leak from some cryptographic operations. Once the TPD is compromised, the attacker could acquire the system’s master private key so that the whole system will be compromised. In order to prevent side-channel attacks, Zhang et al. [21] proposed a novel privacy-preserving authentication scheme. Instead of storing the master private key in the TPD that cannot be updated, their scheme stores security-related information in the TPD, which can be periodically updated. This approach can get rid of the ideal TPD, so it is more practical. However, this scheme uses bilinear mapping and multiple Map-to-Point operations, and thus leads to a heavy computational overhead.

## 3. System Model and Design Goals

In this chapter, we briefly introduce the network model and security requirements. Some notations are defined as shown in Table 1.

### 3.1. Network Model

**Figure 1:**
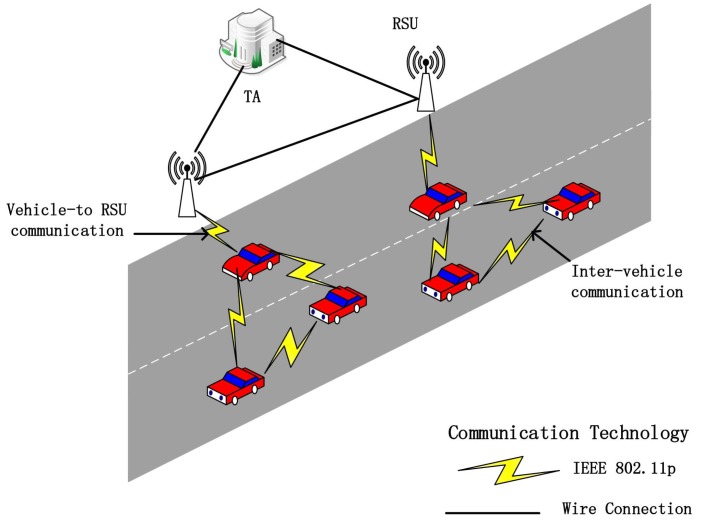
System model.

As shown in Figure 1, an IoV consists of a third-party trusted authority (TA), some RSUs distributed on the roadside and multiple vehicles.

- **TA**: TA is a trusted third party in IoV, with sufficient storage and computing power, and is considered impossible to compromise by an adversary. When an attacker simulates that a vehicle sends a false message, the TA can resume the true identity of the sent message.
- **RSUs**: The RSU is an infrastructure that is distributed on the roadside and communicates with the TA via a wired connection, and communicates with vehicles over a wireless connection to verify the validity of the received message.
- **Vehicles**: Each vehicle is equipped with TPD, and communicates with other vehicles and RSUs through wireless connections. The vehicle periodically broadcasts security-related messages to nearby vehicles and RSUs through the Dedicated Short Range Communications (DSRC) protocol.

### 3.2. Security Requirements

A security scheme for IoV should meet some of the following features:

1. Message integrity: In IoV, we need to ensure that the recipient received the message from the sender, and the message during the sending process has not been modified by the attacker to maintain integrity.
2. Non-forgery: The attacker should not generate a valid signature on behalf of any vehicle under the randomly selected message attack in the random oracle model.
3. Traceability: When an attacker presents as being a legal vehicle and sends a false message that may cause damage, the TA can reveal the real identity of the false message.
4. Non-repudiation: When the trusted third party (TA) retrieves the real identity of the false message, the sender of the message could not deny the attack.
5. Resistance against side-channel attack: The attacker should not be able to obtain any information stored in the tamper-proof device through the side-channel attack.

## 4. The Proposed Scheme

**Figure 2:**
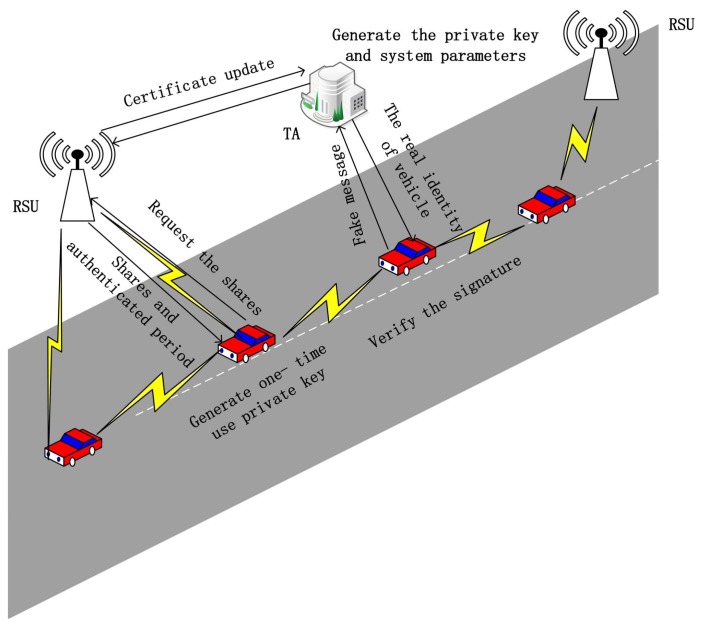
Graphical representation of our scheme.

Recently, some safety-related authentication schemes for IoV have been proposed. However, most of them are heavy in computation and communication, and could not resist some attack existing in IoV. In order to deal with the security problem existing in IoV, we proposed the privacy-preserving authentication using a double pseudonym that does not use bilinear paring, which can be used in the inter-vehicle communications and vehicle to RSU communications. Figure 2 graphically describes the details of our scheme. TA generates the private key and system parameters in our scheme. Each RSU has its own public-private key pairs and the corresponding certification from the TA. When a vehicle enters the range of RSU, then it will request the shares (member secret) of RSU, after authenticating the identity of vehicle via the TA, the shares and the corresponding authenticated period will be sent to the vehicle. This authenticated period is valid for a short time. Once it expires, it should be executed once again. Upon the vehicle receiving the shares and authenticated period, it generates a one-time use private key and signature. This signature could be verified by other vehicles and RSUs. If a vehicle sends a false message, the TA could trace the real identity of the vehicle.

This scheme can be divided into the following modules:

- **System Setup**: In this phase, the TA generates the private key and system parameters.
- **RSU Setup**: In this phase, the RSU can generate its own public-private key and the corresponding certification $cert_{R_{j}}$ from the TA.
- **Vehicle Setup**: In this phase, when the vehicle joins into the IoV, the TA generates the inter-pseudonym identity (IPID). The vehicle chooses the authentication key $\lambda_{i}$, and puts the IPID and the $\lambda_{i}$ into the tamper-proof device.
- **Member key generation**: In this phase, when the vehicle enters the range of the RSU, the vehicle will request to acquire the member secret of the RSU. After the RSU authenticates the identity of the vehicle, it sends the member secret $\left(\beta_{j},\gamma_{j}\right)$ and the corresponding valid period ($VP_{i}$) to the vehicle.
- **Vehicle Sign**: In this phase, if the vehicle wants to send a message, it will first generate its own external pseudonym identity and one-time signing key, and then generate the signature of the corresponding message.
- **Message Verification**: In this phase, we will use batch authentication to verify the validity of signatures without bilinear pairing, which greatly reduced the computation overhead.
- **IPID and authentication key updated**: In this phase, vehicles can use the online mode to update their own inter-pseudonym identity and authentication key and to prevent the attacker from tracking the true identity of the vehicle.

### 4.1. System Setup Phase

In this phase, there are some initialization parameters that preload into the vehicles and RSUs generated by the TA using the following steps. This can be done once, unless the private key of the system is compromised by an attacker, or the system wants to periodically update the system parameters and private key to enhance the system security level:

1. TA selects two large prime *p* and *q* as well as a non-singular elliptic curve *E* defined by the equation $y^{2}=x^{3}+ax+b$ mod *n*, where $a,b\in F_{p}$.
2. TA selects the cyclic addition group *G*, where the *P* is the generator and *q* is the order of group.
3. TA selects a random number $s\in Z_{q}^{*}$ as the secret key of the TA, and calculates $P_{pub}=s\cdot P$ as the public key of the TA.
4. TA selects $E_{\pi }($·)/$D_{\pi }($·) and some hash functions: $h_{1}:$ $G\to Z_{q},$ $h_{2}:$ ${\left\{0,1\right\}}^{*}$ $\to Z_{q},$ $H_{1_{key}}\left(\cdot \right)$: ${\left\{0,1\right\}}^{*}\to {\left\{0,1\right\}}^{l}$, $H_{2}\left(\cdot \right):$ ${\left\{0,1\right\}}^{*}\to \Gamma$, $H_{3}\left(\cdot \right):$ ${\left\{0,1\right\}}^{*}\to {\left\{0,1\right\}}^{l^{\prime }}$, where $H_{1_{key}}\left(\cdot \right)$ as a keyed hash.
5. The system parameters are $\psi =(p,q,a,b,P,P_{pub},h_{1},$ $h_{2}\;,H_{1_{key}}\left(\cdot \right),H_{2}\left(\cdot \right),$ $H_{3}\left(\cdot \right),E_{\pi }\left(.\right)/D_{\pi }\left(.\right))$, putting the system parameters ψ into the vehicles and RSUs in advance.

### 4.2. RSU Setup Phase

In this phase, the RSU generates its own public-private key pairs and the corresponding certification from the TA. This certification can be used only in a short time. Once the period is over, the RSU should execute the step once again. To generate its own public-private key pairs, the RSU randomly chooses two numbers $k_{j},\eta_{j}\in Z_{q}^{*}$ and computes $PK_{R_{j1}}=k_{j}P,PK_{R_{j2}}=\eta_{j}P$. The private key is $\left(k_{j},\eta_{j}\right)$ and the public key is $\left(PK_{R_{j1}},PK_{R_{j2}}\right)$, where $k_{j}$ is used to generate the shares of vehicle, and $\eta_{j}$ is used to generate the secure channel between the RSU and vehicle. After generating its public key, the RSU sends the public key $\left(PK_{R_{j1}},PK_{R_{j2}}\right)$ and its own identity information to the TA through the secure channel. When the TA receives the messages, it generates the certification of RSU, and then the RSU broadcasts the $cert_{R_{j}}$ within its own range.

### 4.3. Vehicle Setup Phase

In this phase, when the vehicle joins the range of the IoV, the information stored in the TPD should be initialized. Assuming the real identity of vehicle is RID, the TA can compute the inter-pseudonym identity $IPID_{V_{i}}=H_{1_{\Lambda }}(RID||VP_{i})$, where the $VP_{i}$ is the valid period of the inter-pseudonym identity like 2 March 2017–3 April 2017. The vehicle chooses the authentication key $\lambda_{i}$, putting the $\psi ,IPID_{V_{i}},\lambda_{i}$ into the TPD. $\left(\mathrm{RID},VP_{i},IPID_{V_{i}},\lambda_{i}\right)$ is stored into the member list ML in the TA.

### 4.4. Member Key Generation Phase

In this phase, a vehicle can obtain the member secrets and the corresponding valid period from the nearest RSU. This process among the vehicle, the RSU and the TA should be confidential. When a vehicle enters the communication range of RSU, the vehicle will receive the certification from RSU, and send the request of acquiring the member secrets and the corresponding valid period of RSU. After the RSU authenticates the identity of the vehicle, it sends the member secret and the valid period to the vehicle. The details are as the following steps:

1. When the vehicle enters the communication range of RSU, the vehicle will receive the certification from RSU and first check the validity of the $cert_{R_{j}}$ that has the format $\left(ID_{R_{j}},\left(PK_{R_{j1}},PK_{R_{j2}},sig_{j}\right)\right)$, where $sig_{j}$ is a signature on $\left(ID_{R_{j}},\left(PK_{R_{j1}},PK_{R_{j2}}\right)\right)$ issued by the TA. If the certification is valid under the public key of the system, extract the public key and identity of RSU from the certification $cert_{R_{j}}$.
2. The vehicle chooses a random number $r\in Z_{q}^{*}$, and computes f=rP, $\pi_{i1}=$$H_{2}(f,PK_{R_{j2}},rPK_{R_{j2}},\;$ $ID_{R_{j}},$$T_{i})$, $\pi_{i2}=$$H_{2}\left(f,P_{pub},\;rP_{pub},\;ID_{R_{j}},T_{i}\right),$ where $T_{i}$ is a timestamp, and $\pi_{i1}$,$\pi_{i2}$ are used as the keys of the symmetric encryption scheme $\left(E_{\pi }\left(.\right)/D_{\pi }\left(.\right)\right)$. Finally, the vehicle computes $p_{j}=E_{\pi_{i2}}\left(\lambda_{i},T_{i}\right)$ and sends $s=\left(f,ID_{R_{j}},p_{j},T_{i}\right)$ to RSU.
3. The RSU receives s from vehicle, if $T_{i}$ is invalid, then it aborts; otherwise, it sends s to the TA through the secure channel. When the TA receives s and first computes $\pi_{i2}=H_{2}\left(f,P_{pub},\mathrm{sf},ID_{R_{j}},T_{i}\right)$, $D_{\pi_{i2}}\left(p_{j}\right)$ to get $\left(\lambda_{i}^{\prime },T_{i}^{\prime }\right)$. If the equation $\lambda_{i}^{\prime }\neq \lambda_{i}$ does not appear in a tuple of the member list $\left(\mathrm{RID},VP_{i},IPID_{V_{i}},\lambda_{i}\right)$ of the TA or $T_{i}\neq T_{i}^{\prime }$ or $VP_{i}$ is invalid, it aborts; otherwise, the TA authenticates the vehicle and sends the authenticated message to RSU.
4. When the RSU receives the authenticated message from the TA, it means the vehicle is legal. RSU first computes $\pi_{i1}=H_{2}\left(f,PK_{R_{j2}},f\eta_{j},ID_{R_{j}},T_{i}\right)$; and chooses an authenticated period $\tau_{p}$ and member secret $\left(\beta_{j},\gamma_{j}\right)$, where $\beta_{j}$ and $\gamma_{j}$ satisfy $k_{j}=\beta_{j}\cdot \gamma_{j}$; it computes $h_{R_{j}}=$$H_{1_{\pi_{i1}}}\left(\beta_{j},\gamma_{j},\tau_{p}\right)$, and $p_{j}^{\prime }=E_{\pi_{i1}}\left(\beta_{j},\gamma_{j},\tau_{p},h_{R_{j}}\right)$; and sends $t=(H_{3}\left(f\right),$$p_{j}^{\prime })$ to the vehicle.
5. When the vehicle receives the *t* and $D_{\pi i1}\left(p_{j}^{\prime }\right)$ to get $\left(\beta_{j},\gamma_{j},\tau_{p},h_{R_{j}}\right)$, it verifies whether the equation $h_{R_{j}}=H_{1_{\pi_{i1}}}\left(\beta_{j},\gamma_{j},\tau_{p}\right)$ holds. If so, it lets the member secret and authenticated period in the TPD; otherwise, it aborts. This member key can only be used under the authenticated period, and, once it expires, the member key stored in the TPD is deleted.

### 4.5. Vehicle Signature Phase

In this phase, when a vehicle obtains the member secret $\left(\beta_{j},\gamma_{j}\right)$ from the RSU and the validity period of member secret is within the authorized period, the vehicle will generate the external pseudonym identity of the vehicle and the digital signature of the message. Finally, a vehicle broadcasts the external pseudonym identity, the message as well as the digital signature to other vehicles and the RSU. The details are as the following steps:

1. Vehicle computes the external pseudonym identity $PPID_{i}=H_{3}\left(IPID_{V_{i}},T_{i}\right)$ and the one time signature key $sk_{i}=\left(\beta_{j}\cdot \gamma_{j}\right)\cdot h_{1}\left(PPID_{i}\right)modn$.
2. The vehicle chooses a random number $r_{i}\in Z_{q}^{*}$, and computes $R_{i}=r_{i}\cdot P$, $\beta_{i}=$$h_{2}\left(PPID_{i}\left|\left|R_{i}\right|\right|M_{i}\right)$, $S_{i}=$$sk_{i}+\beta_{i}\cdot r_{i}$. Then the vehicle sends $\left(M_{i},PPID_{i},R_{i},S_{i}\right)$ to nearby vehicles and RSUs.
3. The member secret $\left(\beta_{j},\gamma_{j}\right)$ stored in the TPD needs to be periodically updated. Choose a random number $r\in Z_{q}^{*}$, and set $\beta_{j}=r\cdot \beta_{j}$, $\gamma_{j}=r\cdot \gamma_{j}$ as the new member secret.

### 4.6. Message Verification Phase

This phase allows the verifier to check the validity of the received message without bilinear pairing, which greatly reduces the computation overhead. Moreover, our scheme can support the batch verification function, which can verify many messages at the same time to improve performance. Then, we will show the details of verifying a single message and many messages.

- **Single message verification**: When the verifier receives the safety-related message $\left(M_{i},PPID_{i},R_{i},S_{i}\right)$ broadcasted from the vehicle, it could use the system parameters ψ to verify the validity of the message. The details are as following:
  1. The verifier first checks the validity of timestamp $T_{i}$. If $T_{i}$ is invalid, it aborts; otherwise, it executes the next step.
  2. The verifier checks whether the equation $S_{i}\cdot P=h_{1}\left(PPID_{i}\right)\cdot PK_{R_{j1}}+\beta_{i}\cdot R_{i}$ holds. If it holds, the verifier receives the message; otherwise, it rejects the message.
  Due to $sk_{i}=(\beta_{j}\cdot \gamma_{j})\cdot h_{1}\left(PPID_{i}\right)modn$, $\beta_{j}\cdot \gamma_{j}=k_{j}$, $PK_{R_{j1}}=k_{j}\cdot P$, $R_{i}=r_{i}\cdot P$, $\beta_{i}=h_{2}\left(PPID_{i}\left|\left|R_{i}\right|\right|M_{i}\right)$ and $S_{i}=sk_{i}+\beta_{i}\cdot r_{i}$, we can check the equation through the following steps:
  (1) $$\begin{array}{c} S_{i}\cdot P=\left(sk_{i}+\beta_{i}r_{i}\right)\cdot P\\ =\left(\left(\beta_{j}\cdot \gamma_{j}\right)\cdot h_{1}\left(PPID_{i}\right)+\beta_{i}\cdot r_{i}\right)\cdot P\\ =\left(\beta_{j}\cdot \gamma_{j}\right)\cdot h_{1}\left(PPID_{i}\right)\cdot P+\beta_{i}\cdot r_{i}\cdot P\\ =h_{1}\left(PPID_{i}\right)\cdot PK_{R_{j1}}+\beta_{i}\cdot R_{i}. \end{array}$$
  Hence, the correctness of the single message verification is verified.
- **Multiple messages batch verification**: We used a small index test technique during the batch verification procedure to ensure the non-repudiation of the signature. A vector, including the small random integer, is used to detect the modification of the batch signature in the small index test technique. After receiving multiple messages $\left(M_{1},PPID_{1},R_{1},S_{1}\right)$, $\left(M_{2},PPID_{2},R_{2},S_{2}\right)$, ...,$\left(M_{n},PPID_{n},R_{n},S_{n}\right)$, a verifier uses the system parameter to verify the validity of the many messages at the same time. The details are as the following steps:
  1. Verifier first checks the validity of $T_{i}$, where i=1,2,…,n. If $T_{i}$ is invalid, the verifier rejects the messages; otherwise, it executes the next step.
  2. Verifier chooses a random vector $v=\{v_{1},v_{2},\ldots ,$$v_{n}\}$, where $v_{i}$ is a small random integer in $\left[1,2^{t}\right]$ and *t* is a small integer with low overhead. Then, the verifier checks the correctness of the equation $(\sum_{i=1}^{n}v_{i}\cdot S_{i})\cdot P=\sum_{i=1}^{n}(V_{i}\cdot h_{1}\left(PPID_{i}\right))\cdot PK_{R_{j1}}+\sum_{i=1}^{n}(v_{i}\cdot \beta_{i}\cdot R_{i})$. If it does not hold, the verifier rejects the messages; otherwise, the verifier receives the messages.
  Due to $sk_{i}=\left(\alpha_{j}\cdot \beta_{j}\right)\cdot h_{1}\left(PPID_{i}\right)modn$, $\beta_{j}\cdot \gamma_{j}=k_{j}$, $PK_{R_{j1}}=k_{j}\cdot P$, $R_{i}=r_{i}\cdot P$, $\beta_{i}=h_{2}\left(PPID_{i}\left|\left|R_{i}\right|\right|M_{i}\right)$ and $S_{i}=sk_{i}+\beta_{i}\cdot r_{i}$, we can check the equation through the following steps:
  (2) $$\begin{array}{c} \left(\sum_{i=1}^{n}v_{i}\cdot S_{i}\right)\cdot P=\left(\sum_{i=1}^{n}v_{i}\left(sk_{i}+\beta_{i}\cdot r_{i}\right)\right)\cdot P\\ =\left(\sum_{i=1}^{n}v_{i}\left(\left(\beta_{j}\cdot \gamma_{j}\right)\cdot h_{1}\left(PPID_{i}\right)+\beta_{i}\cdot r_{i}\right)\right)\cdot P\\ =\sum_{i=1}^{n}\left(v_{i}\cdot \left(\left(\beta_{j}\cdot \gamma_{j}\right)\cdot h_{1}\left(PPID_{i}\right)\cdot P+\beta_{i}\cdot r_{i}\cdot P\right)\right)\\ =\sum_{i=1}^{n}\left(v_{i}\cdot h_{1}\left(PPID_{i}\right)\cdot PK_{R_{j1}}+v_{i}\cdot \beta_{i}\;\cdot R_{i}\right)\\ =\sum_{i=1}^{n}\left(v_{i}\cdot h_{1}\left(PPID_{i}\right)\right)\cdot PK_{R_{j1}}+\sum_{i=1}^{n}\left(v_{i}\cdot \beta_{i}\cdot R_{i}\right)\\ =\sum_{i=1}^{n}\left(v_{i}\cdot h_{1}\left(PPID_{i}\right)\right)\cdot PK_{R_{j1}}+\sum_{i=1}^{n}\left(v_{i}\cdot \beta_{i}\cdot R_{i}\right). \end{array}$$

Hence, the correctness of the multiple messages verification is verified.

### 4.7. IPID and Authentication Key Update Phase

At this phase, the vehicle can use the online model to update the internal pseudo-identity and authentication key stored in the TPD. The details are as following:

1. When a vehicle wants to update the internal pseudo-identity and authentication key, it first chooses a random number $t\in Z_{q}^{*}$, and computes g=t·P, $\pi_{i}=H_{2}\left(g,P_{pub},tP_{pub},T_{i}\right)$, $p_{i}=$$E_{\pi_{i}}\left(\lambda_{i},T_{i}\right)$. Then, it sends $z=\left(g,T_{i},p_{i}\right)$ to the TA through the nearby RSU.
2. The TA receives *z* and checks the validity of $T_{i}$. If $T_{i}$ is invalid, it aborts; otherwise, it executes the following steps:
  - TA computes $\pi_{i}=H_{2}(g,P_{pub},s$·$g,T_{i})$ and $D_{\pi_{i}}$$\left(p_{i}\right)$ to get ($\lambda_{i}^{\prime },T_{i}^{\prime }$).
  - TA checks the validity of $T_{i}^{\prime }$, if $T_{i}^{\prime }$ is invalid, it aborts; otherwise, it executes the next step.
  - TA searches the member list for a tuple ($\mathrm{RID},VP_{i},$ $IPID_{V_{i}},\lambda_{i})$ such as $\lambda_{i}=\lambda_{i}^{\prime }$. If such a tuple does not exist, it aborts; otherwise, it executes the next step.
  - TA checks the validity of $VP_{i}$. If it is invalid, choose a new valid period $V{P_{i}}^{\prime }$. TA computes $IPID_{V_{i}}^{\prime }=$ $H_{1_{\Lambda }}(RID||V{P_{i}}^{\prime })$ and chooses a new authentication key $\hat{\lambda_{i}}$; otherwise, it aborts.
  - TA computes $p_{i}=E_{\pi_{i}}\left(IPID_{V_{i}}^{\prime },\hat{\lambda_{i}},T_{i}^{\prime },h_{TA}\right)$. If $h_{TA}=H_{1_{\lambda_{i}^{\prime }}}(IPID_{V_{i}}^{\prime },\hat{\lambda_{i}},T_{i})$ is an HAMC, sends $\left(H_{3}(g),p_{i}^{\prime }\right)$ to the vehicle and put $(\mathrm{RID},V{P_{i}}^{\prime },$$IPID_{V_{i}}^{\prime },\hat{\lambda_{i}})$ into ML.
3. After a vehicle receives $\left(H_{3}(g),p_{i}^{\prime }\right)$, it first computes $D_{\pi_{i}}(p_{i}^{\prime })$ to get $\left(IPID_{V_{i}}^{\prime },\lambda_{i}^{\prime },T_{i}^{\prime },{h_{TA}}^{\prime }\right)$. Then, the vehicle checks the validity of $T_{i}^{\prime }$ and ${h_{TA}}^{\prime }$. If it is invalid, set the $\left(IPID_{V_{i}}^{\prime },\lambda_{i}^{\prime }\right)$ as the new internal pseudo-identity and authentication key.

## 5. Security Proof and Analysis

In this section, because it is difficult to address the computational Elliptic Curve Discrete Logarithm (ECDL) problem, we prove that the proposed identity-based scheme has the feature of non-forgery. In addition, we also show that our scheme can satisfy the security requirement and illustrate the differences between our scheme and others.

### 5.1. Security Analysis

In this sub-section, we will show that an attacker could not generate a valid signature on behalf of any vehicle through the game that is made up of a challenger *C* and an adversary *A*.

**Definition** **1.** *Since it is difficult to address the computational Elliptic Curve Discrete Logarithm (ECDL) problem, the proposed scheme is security existential forgery under the randomly selected message attack in the random oracle model. The proof is as follows.*

**Theorem** **1.** *Our scheme for IoV is secure in the random oracle.*

Assuming there is an adversary that could forge message $\left(M_{i},PPID_{i},R_{i},S_{i}\right)$, then we construct a challenger *C*, which could solve the ECDL problem through running *A* as a subroutine. The details are as the following steps:

Setup stage: Challenger *C* first sets $Q=PK_{R_{j1}}$, then it sends the system parameters $\psi =(p,q,a,b,P,P_{pub},$ $h_{1},$ $h_{2}\;,H_{1_{key}}\left(\cdot \right),H_{2}\left(\cdot \right),H_{3}\left(\cdot \right),E_{\pi }\left(.\right)/D_{\pi }\left(.\right))$ to an adversary *A*.

**$h_{1}-oracle$**: Challenger *C* first initializes the list $L_{h_{1}}$ with the form of $\left(\langle PPID_{i},\tau_{h_{1}}\rangle \right)$. When receiving the query of the message with the form of $<PPID_{i}>$ from the adversary *A*, the challenger *C* checks a tuple of the $<PPID_{i}>$ to find out whether it appears in the list $L_{h_{1}}$. If the tuple exists in the list $L_{h_{1}}$, then send $\tau_{h_{1}}=h_{1}\left(PPID_{i}\right)$ to the adversary *A*; otherwise, *C* chooses a random number $\tau_{h_{1}}\in Z_{q}^{*}$ and sets the tuple $\langle PPID_{i},\tau_{h_{1}}\rangle$ into the $L_{h_{1}}$, finally sending the $\tau_{h_{1}}=$ $h_{1}\left(PPID_{i}\right)$ to *A*.

$h_{2}-oracle$: Challenger *C* first initializes the list $L_{h_{2}}$ with the form of $L_{h_{2}}$$\left(\langle PPID_{i},R_{i},M_{i},\tau_{h_{2}}\rangle \right)$. When receiving the query of the message with the form of $\langle PPID_{i},R_{i},M_{i}\rangle$ from the adversary *A*, the challenger *C* checks a tuple of the $\langle PPID_{i},R_{i},M_{i}\rangle$ for whether it appears in the list $L_{h_{2}}$. If the tuple exists in in the list $L_{h_{2}}$, then sends $\tau_{h_{2}}=h_{2}\left(PPID_{i}\left|\left|R_{i}\right|\right|M_{i}\right)$ to the adversary *A*; otherwise, *C* chooses a random number $\tau_{h_{2}}\in Z_{q}^{*}$ and sets the tuple $\left(\langle PPID_{i},R_{i},M_{i},\tau_{h_{2}}\rangle \right)$ into the $L_{h_{2}}$, and finally sends the $\tau_{h_{2}}=h_{2}\left(PPID_{i}\left|\left|R_{i}\right|\right|M_{i}\right)$ to *A*.

sign-oracle: Upon receiving the message $M_{i}$ from an adversary *A*, challenger *C* generates random numbers $S_{i},$ $h_{i,1},\beta_{i}\in Z_{q}^{*}$ and $PPID_{i}$. Challenger *C* puts $\langle PPID_{i},h_{i,1}\rangle$ and $\left(M_{i},PPID_{i},R_{i},S_{i}\right)$ to the adversary *A*, and it is easy to verify that the equation $S_{i}\cdot P=h_{1}\left(PPID_{i}\right)\cdot PK_{R_{j1}}+\beta_{i}\cdot R_{i}$ holds. Therefore, the message and signature $\left(M_{i},PPID_{i},R_{i},S_{i}\right)$, which *A* acquired from the inquiry from *C*, are valid.

Output: Finally, *A* outputs the message $(M_{i},PPID_{i},R_{i},$ $S_{i})$. *C* checks whether the equation holds:

(3) $$\begin{array}{c} S_{i}\cdot P=h_{1}\left(PPID_{i}\right)\cdot PK_{R_{j1}}+\beta_{i}\cdot R_{i}. \end{array}$$

If it does not hold, *C* aborts the process; otherwise, because of the forged lemma, if *A* executes $h_{1}-oracle$ once again, a valid message $\left(M_{i},PPID_{i},R_{i},S_{i}^{\prime }\right)$ will be generated. It can also conclude the similar equation:

(4) $$\begin{array}{c} S_{i}^{\prime }\cdot P={\left(h_{1}\left(PPID_{i}\right)\right)}^{\prime }\cdot PK_{R_{j1}}+\beta_{i}\cdot R_{i}. \end{array}$$

According to Equations (3) and (4), we could get

(5) $$\begin{array}{c} \left(S_{i}-S_{i}^{\prime }\right)\cdot P=S_{i}\cdot P-S_{i}^{\prime }\cdot P\\ =h_{1}\left(PPID_{i}\right)\cdot PK_{R_{j1}}+\beta_{i}\;\cdot R_{i}\\ -\left({\left(h_{1}\left(PPID_{i}\right)\right)}^{\prime }\cdot PK_{R_{j1}}+\beta_{i}\cdot R_{i}\right)\\ =\left(h_{1}\left(PPID_{i}\right)-{\left(h_{1}\left(PPID_{i}\right)\right)}^{\prime }\right)\cdot PK_{R_{j1}}\\ =\left(h_{1}\left(PPID_{i}\right)-{\left(h_{1}\left(PPID_{i}\right)\right)}^{\prime }\right)\cdot k_{j}\cdot P \end{array}$$

and

(6) $$\begin{array}{c} \left(S_{i}-{S_{i}}^{\prime }\right)=\left(h_{1}\left(PPID_{i}\right)-{\left(h_{1}\left(PPID_{i}\right)\right)}^{\prime }\right)\cdot k_{j}. \end{array}$$

Therefore, *C* outputs the ${\left(h_{1}\left(PPID_{i}\right)-{\left(h_{1}\left(PPID_{i}\right)\right)}^{\prime }\right)}^{-1}$·$\left(S_{i}-S_{i}^{\prime }\right)$. However, it is difficult to address the computational Elliptic Curve Discrete Logarithm (ECDL) problem, and the security of the proposed scheme is secure against forgery under the randomly selected message attack in the random oracle model.

1. **Message integrity**: According to Theorem 1, because it is difficult to solve the ECDL problem, the signature used in our scheme is not forged under the random oracle model. Therefore, no adversary can simulate a legal vehicle to generate a valid signature or modify a legal signature. We can verify the equation that $S_{i}\cdot P=h_{1}\left(PPID_{i}\right)\cdot PK_{R_{j1}}+\beta_{i}\cdot R_{i}$ holds to check the validity and integrity of the message $\left(M_{i},PPID_{i},R_{i},S_{i}\right)$ . Thus, the proposed scheme can achieve message integrity.
2. **Non-forgery**: Since it is difficult to address the computational Elliptic Curve Discrete Logarithm (ECDL) problem, the attacker could not generate a valid signature on behalf of any vehicle under the randomly selected message attack in the random oracle model. Thus, the proposed scheme can achieve non-forgery.
3. **Traceability**: During this stage, when the adversary sends false messages which cause damage, the TA can trace the real identity of the corresponding message. Assuming the public pseudonym identity of the vehicle is $PPID_{i}$, the TA can extract the timestamp from the message $M_{i}$, which can find the valid period $VP_{i}$ of the internal pseudo-identity of vehicle. Then, the TA can verify whether the equation $H_{3}\left(IPID_{V_{i}},T_{i}\right)$ = $PPID_{i}$ holds, where the $IPID_{V_{i}}$ is in the tuple of member list $\left(\mathrm{RID},VP_{i},IPID_{V_{i}},\lambda_{i}\right)$. If it holds, the TA outputs the real identity of vehicle.
4. **Non-repudiation**: Once the TA traces the real identity of false message, the message sender could not deny that he has sent this false message. To achieve this goal, in our scheme, we use a random vector $v=\left\{v_{1},v_{2},\ldots ,v_{n}\right\}$ to ensure an attacker cannot deny its signature in a message sent by exchanging signatures among several different messages.
5. **Resistance side channel attack**: In this paper, we use the more realistic TPD to resist side channel attack. There are three types of related information (IPID, authentication key, and member secret) stored in the TPD of our scheme. Due to the first type of secret often being used, if the vehicle does not periodically update this information, it will give the attacker a chance to recover the real identity of vehicle. In our scheme, before the attacker can probe the related information to recover the IPID through the side channel attack, the IPIP has already been updated. Secondly, the authenticated key can only be used during the authentication of vehicle. It is much harder for the attacker to resume the authenticated key than recover the IPID. In addition, as for the member secret, even if the adversary could recover the member secret, only the vehicle in the nearby RSU can be influenced. Furthermore, because the RSU can periodically update its public-private key pairs, the attacker could not acquire enough information through the side channel to resume the member key stored in the TPD.

### 5.2. Security Comparison

In this sub-section, we compare the proposed scheme with other existing schemes in terms of satisfactory security requirements. The comparison results are summarized in Table 2, where sr1, sr2, sr3, sr4, sr5 denote the message integrity, non-forgery, traceability, non-repudiation and resistance side channel attack, respectively.

As shown in Table 2, we can conclude that the schemes of Zhang et al. [18], Bayat et al. [19], and He et al. [20] could not satisfy all five of the security requirements. However, our scheme could satisfy all security requirements.

## 6. Performance Analysis and Comparison

In this section, we will analyze the proposed identity-based scheme compared with other related schemes in terms of the computation overhead and communication overhead.

### 6.1. Computation Overhead Analysis

In the scheme-based bilinear pairing proposed by Zhang et al. [18] and Bayat et al. [19], the order *q* of group *G* in the bilinear pairing e: $G\times G\to G_{T}$, generated by the Elliptic Curve $y^{2}=x^{3}+x$ mod *n* to achieve the security level of 80 bits, where *n* is the 512-bit prime number and the order *q* of the group *G* is a 160-bit prime number. However, among the schemes based on the Elliptic Curve, such as the scheme proposed by He et al. [20], the order *q* of group *G* is generated by the Elliptic Curve $y^{2}=x^{3}+ax+b$ mod *n* to achieve the same security level compared with the scheme based on the bilinear pairing, where *n* and *q* are the 160-bit prime numbers. For convenience, some time-consuming cryptographic operations [22] are defined as follows: $T_{p}$ denotes the execution time of the bilinear pairing operation; $T_{mp-p}$ denotes the execution time of the small scale multiplication operation; $T_{mtp}$ denotes the execution time of a Map-to-Point operation; and $T_{mp-ECC}$ denotes the execution time of the small scale multiplication operation based on the Elliptic Curve. Table 3 lists the execution time required for these operations [20].

The computation overhead of our scheme can be compared with other schemes in three aspects, letting PSGH and SMVH and MMVH denote the pseudonym and signature generation phase, signal message verification phase and multiple messages verification phase, respectively. Details are only shown in Zhang et al.’s scheme [21] and our scheme. The other schemes can be analyzed by the same method. Table 4 lists the computation overhead of our scheme compared with the schemes of Zhang et al. [18], Bayat et al. [19], Zhang et al. [21] and He et al. [20].

In the pseudonym and signature generation phase of Zhang et al’s scheme [21], which needs to execute the two Map-to-Point operations, the whole computation time of this phase is $2T_{mtp}=8.812$ ms. During the signal message verification phase, there are two bilinear pairing operations and two Map-to-Point operations that need to be executed. Thus, the whole computation time of this phase is $2T_{p}+2T_{mtp}=17.234$ ms. In the multiple messages verification phase, there are two bilinear pairing operations and 2n Map-to-Point operations that need to be executed. Thus, the whole computation time of this phase is $2T_{p}+2T_{mtp}=8.812n+8.422$ ms.

**Figure 3:**
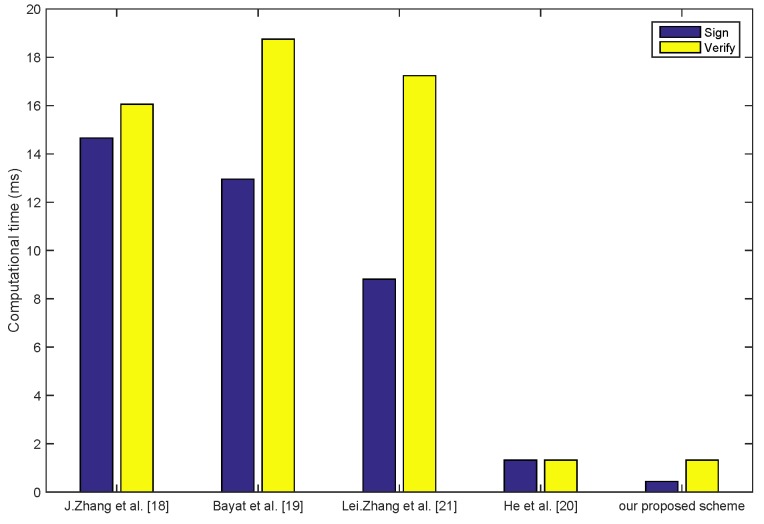
Computation overhead comparison of signing and verifying a single message.

**Figure 4:**
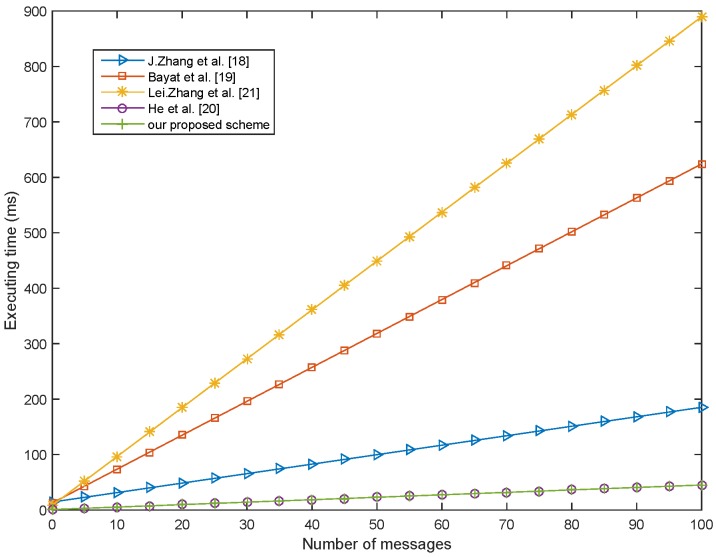
Computation overhead comparison of verifying multiple message.

In the pseudonym and signature generation phase of our scheme, which needs to execute a small scale multiplication operation based on the Elliptic Curve, the whole computation time of this phase is $T_{mp-ECC}=0.442$ ms. During the signal message verification phase, there are three small scale multiplication operations based on the Elliptic Curve that need to be executed. Thus, the whole computation time of this phase is $3T_{mp-ECC}=1.326$ ms. In the multiple message verification phase, there are (n+2) small scale multiplication operation based on the Elliptic Curve that need to be executed. Thus, the whole computation time of this phase is $\left(n+2\right)T_{mp-ECC}=0.442\left(n+2\right)$ ms. Figure 3 shows the computation overhead to sign and verify the single message in each scheme. Compared with the schemes of Bayat et al. [19] and Zhang et al. [21] using the bilinear pairing, our scheme’s computation is lower. At the same time, our scheme is also lower than the scheme of He et al. [20] in terms of computation. Figure 4 shows the total execution time of the batch verification as the amount of the vehicle increasing in each scheme. When the authenticated vehicle is increased to 100, in our scheme, the total execution time is less than 50 ms. Hence, our scheme is more suitable for the scene of multiple vehicles in IoV.

### 6.2. Communication Overhead Analysis

In this section, the communication overheads of our scheme compared with other schemes will be analyzed. In the group $G_{1}$ based on bilinear mapping, the size of the elements in $G_{1}$ is 64 × 2 = 128 byte [23]. However, in the group *G* based on the Elliptic Curve, the size of the elements in $G_{1}$ is 20 × 2 = 40 byte. Furthermore, we assume that the size of result of the general hash function is 20 bytes and the size of the timestamp is 4 bytes [24]. In addition, we do not consider the size of the message that is transmitted by the vehicle in this phase [25]. Table 5 lists the computation overheads of our scheme compared with other schemes.

In Table 5, the communication message is $\{AID_{i},M_{i},$$S_{i},T_{i}\}$ in Zhang et al.’s scheme [18], where $AID_{i}=\left\{AID_{1},AID_{2}\right\}$$,\;AID_{1},AID_{2},S_{i}\in G_{1}$, hence the communication overhead of sending single message is 128 × 3 + 4 = 388 bytes. When multiple messages are broadcasted, which needs n pseudonym, signature and timestamp, the total communication overhead of sending multiple messages is 388n bytes. In addition, in He et al.’s scheme [20], due to the $AID_{1},$$AID_{2},R_{i}\in G,\sigma_{i}\in Z_{q}^{*}$, $T_{i}$ is a timestamp, and the communication overhead of sending a single message and multiple messages are 40 × 3 + 20 + 4 = 144 byte and 144 n bytes, respectively. The other scheme’s communication overhead can be concluded by the same method. In our scheme, the communication messages include $PPID_{i}\in Z_{q}^{*}$, $R_{i}\in G,S_{i}\in Z_{q}^{*}$, whose overhead is 20 × 2 + 40 = 80 byte. The communication overhead of sending multiple messages is 80n bytes.

## 7. Conclusions

In this paper, we propose a privacy-preserving authentication scheme using a double pseudonym that supports both Vehicle to Vehicle (V2V) communication and Vehicle to Infrastructure (V2I) communication in IoV. Firstly, unlike other schemes, which store the system master secret (that cannot be updated) in the TPD, in our scheme, the information stored in the TPD is regularly updated. Therefore, the proposed scheme can resist side-channel attacks and hence is more practical. Secondly, the security analysis shows that our scheme can satisfy the security requirements for IoV. Furthermore, performance analysis and comparison show that our scheme is better than other schemes in terms of computation overhead and communication overhead. This shows that our scheme is more suitable used in the IoV.

As for future work, we will pay more attention to addressing the extreme environment in which the system suffers Denial of Service (DoS) attack during the messages broadcast. Since the Dos attack is hard to defend and causes huge damage in the batch verification, addressing the DoS attack has become an urgent task in future research. 

## Figures and Tables

**Table 1:** List of notations and definitions.

Notation	Definitions
TA	A trusted authority
$R_{j}$	The *j*-th RSU
$V_{i}$	The *i*-th vehicle
$s,P_{pub}$	the private key and public key of TA
$cert_{R_{j}}$	A certificate of $R_{j}$ issued by TA
$ID_{\left\{R_{j},V_{i}\;\right\}}$	The real identity of $R_{j}$ or $V_{i}$
$VP_{i}$	The validity period of IPID
$IPID_{V_{i}}$	An internal pseudonym identity of $V_{i}$, generated by the TA based on $ID_{V_{i}}$
$PPID_{i,t}$	The public pseudonym identity of $V_{i}$, generated from $IPID_{V_{i}}$ of $V_{i}$
$h_{\left\{R_{j}\;,\mathrm{TA}\right\}}$	A hash-based message authentication code generated by $R_{j}$ or the TA
$E_{\pi }\left(.\right)/D_{\pi }\left(.\right)$	A symmetric encryption scheme, where π is the key

**Table 2:** The security comparisons of each scheme.

	sr1	sr2	sr3	sr4	sr5
Zhang et al. [18]	*√*	×	*√*	*√*	×
Bayat et al. [19]	*√*	×	*√*	*√*	×
Zhang et al. [21]	*√*	*√*	*√*	*√*	*√*
He et al. [20]	*√*	*√*	*√*	*√*	×
Our Scheme	*√*	*√*	*√*	*√*	*√*

**Table 3:** Different execution time of each cryptographic operations.

Cryptographic Operation	Execution Time
$T_{p}$	4.211 ms
$T_{mp-p}$	1.709 ms
$T_{mtp}$	4.406 ms
$T_{mp-ECC}$	0.442 ms

**Table 4:** The computation overhead of each scheme.

Scheme	PSGH	SMVH	MMVH
Zhang et al. [18]	$6T_{mp-tp}$ $+T_{mtp}\;\approx$ 14.66 ms	$3T_{bp}+$ $2T_{mp-bp}$ ≈16.051 ms	$\left(n+1\right)T_{mp-bp}+$ $3T_{bp}\approx 12.633+$ 1.709 (n + 1) ms
Bayat et al. [19]	$5T_{mp-bp}$ $+T_{mtp}\approx$ 12.951 ms	$3T_{bp}+T_{mtp}$ $+T_{mp-bp}\approx$ 18.748 ms	$3T_{bp}+nT_{mp-bp}$ $+nT_{mtp}\approx 6.115n$ +12.633 ms
Zhang et al. [21]	$2T_{mtp}\approx$ 8.812 ms	$2T_{bp}+2T_{mtp}$ ≈17.234 ms	$2T_{bp}+2nT_{mtp}\;\approx$ 8.812n+8.422 ms
He et al. [20]	$3T_{mp-ECC}$ ≈1.326 ms	$3T_{mp-ECC}$ ≈1.326 ms	$\left(n+2\right)T_{mp-ECC}$ $\approx 0.442\left(n+2\right)$ ms
Our Scheme	$T_{mp-ECC}$ ≈0.442 ms	$3T_{mp-ECC}$ ≈1.326 ms	$\left(n+2\right)T_{mp-ECC}$ $\approx 0.442\left(n+2\right)$ ms

**Table 5:** The communication overhead of each scheme.

Scheme	Sending a Single Message	Sending n Messages
Zhang et al. [18]	388 bytes	388 n bytes
Bayat et al. [19]	388 bytes	388 n bytes
Zhang et al. [21]	148 bytes	148 n bytes
He et al. [20]	144 bytes	144 n bytes
Our Scheme	80 bytes	80 n bytes
